# RNA Secondary Structures with Limited Base Pair Span: Exact Backtracking and an Application

**DOI:** 10.3390/genes12010014

**Published:** 2020-12-24

**Authors:** Ronny Lorenz, Peter F. Stadler

**Affiliations:** 1 Institute for Theoretical Chemistry, University of Vienna, Währingerstraße 17, A-1090 Vienna, Austria; 2 Bioinformatics Group, Department of Computer Science, and Interdisciplinary Center for Bioinformatics, German Centre for Integrative Biodiversity Research (iDiv) Halle-Jena-Leipzig, Competence Center for Scalable Data Services and Solutions, and Leipzig Research Center for Civilization Diseases, University of Leipzig, Härtelstraße 16-18, D-04107 Leipzig, Germany; 3 Max Planck Institute for Mathematics in the Sciences, Inselstraße 22, D-04103 Leipzig, Germany; 4 Facultad de Ciencias, Universidad National de Colombia, Sede Bogotá 111321, Colombia; 5 Santa Fe Institute, 1399 Hyde Park Rd., Santa Fe, NM 87501, USA

**Keywords:** RNA secondary structure prediction, scanning algorithm, hyper-stable RNA elements

## Abstract

The accuracy of RNA secondary structure prediction decreases with the span of a base pair, i.e., the number of nucleotides that it encloses. The dynamic programming algorithms for RNA folding can be easily specialized in order to consider only base pairs with a limited span *L*, reducing the memory requirements to O(nL), and further to O(n) by interleaving backtracking. However, the latter is an approximation that precludes the retrieval of the globally optimal structure. So far, the ViennaRNA package therefore does not provide a tool for computing optimal, span-restricted minimum energy structure. Here, we report on an efficient backtracking algorithm that reconstructs the globally optimal structure from the locally optimal fragments that are produced by the interleaved backtracking implemented in RNALfold. An implementation is integrated into the ViennaRNA package. The forward and the backtracking recursions of RNALfold are both easily constrained to structural components with a sufficiently negative *z*-scores. This provides a convenient method in order to identify hyper-stable structural elements. A screen of the *C. elegans* genome shows that such features are more abundant in real genomic sequences when compared to a di-nucleotide shuffled background model.

## 1. Introduction

Long-range base pairs are notoriously difficult to predict in RNA structures. The main reasons are that parts of the folding process in very long RNAs, say beyond a few hundred nucleotides, are likely to be influenced by co-transcriptional folding, and that RNAs are rarely, if ever, isolated in the cell. Consequently, long-range base pairs often do not fold as predicted by thermodynamic folding rules alone [1,2,3]. Performance limitations are also a consideration for very long sequences, since the effort grows cubicly with the sequence length *n*. Several tools have become available, which restrict the span of base pairs (k,l) to l−k+1≤L, including RNALfold [4], Rfold [5], and LocalFold [6]. An alternative approach penalizes long-range base pairs by reducing their energy contribution [2]. The two ideas were combined in [3]. Here, a sigmoidal function is used in order to interpolate between the full energy parameters and an upper bound on the base pair span. Restrictions on the base pair span are easily incorporated into the dynamic programming recursions [4,5,6]. This has the added benefit of resulting in an asymptotically linear resource consumption, namely $O(L^{2}n)$ time and O(Ln) memory in terms of the sequence length *n* and the maximum base pair span *L*. The main memory requirement can be reduced to $O(n+L^{2})$ by writing intermediate results to the disk. This makes it possible to scan an entire genome for local secondary structure elements.

In this contributionm we first close a gap in the implementation of the ViennaRNA package [7,8]. The RNALfold program provides a tool for computing minimum free energy structure with limited span in $O(n+L^{2})$ memory, but it only produces local candidate structures. Here, we show that these local structures can be assembled efficiently, in O(nL) time, in order to yield the global minimum energy structure. We then discuss an additional restriction to unexpectedly stable local secondary structures, and, finally, sketch some application scenarios.

## 2. Theory

### 2.1. Backtracking from External Memory

Consider the problem of folding RNA structure with a maximal base pair span *L*, i.e., for every base pair (i,j) holds j−i+1≤L. In the following, we write $D_{kl}$ for the optimal sub-structure on the sequence interval [k,l] subject to the additional condition that the interval contains a single component, i.e., a substructure that is enclosed by base pair that itself is not contained inside any other base pair. Furthermore, we write $C_{kl}$ for the minimal free energy of a structure that is enclosed by the base pair (k,l). In order to accomodate the so-called dangling ends appearing in the standard (Turner) energy model [9], we set

(1) $$D_{kl}=\min\left\{C_{kl},C_{k+1,l}+d^{5^{\prime }}\left(k\right),C_{k,l-1}+d^{3^{\prime }}\left(l\right),C_{k+1,l-1}+d^{*}\left(k,l\right)\right\}$$

Here, $d^{5^{\prime }}\left(\;.\;\right)$, $d^{3^{\prime }}\left(\;.\;\right)$, and $d^{*}\left(\;.\;\right)$ denote the 5’- and 3’- dangle parameters, and the dangling mismatch energy contributions, resp. The recursions that involve $D_{kl}$ correspond to an ambiguous decomposition of the secondary structure. Thus they can be used for energy minimization, but they cannot be translated directly for probabilistic models and partition function calculations. In the absence of dangling end contributions, we may use $D_{kl}=C_{kl}$.

The basic idea of RNALfold [4] can be summarized, as follows: denote, by $f_{k}$, the optimal free energy of a secondary structure with maximal base pair span *L* on the interval [k,n]. This quantity satisfies the recursion

(2) $$f_{k}=\min\left\{\begin{array}{c} f_{k+1}\\ \min_{k<l\leq k+L-1}\left[D_{kl}+f_{l+1}\right] \end{array}\right.$$

The first alternative shown in Equation (2) corresponds to *k* being unpaired (and not subject to a dangling end contribution). The second alternative corresponds to a structure beginning with a single component structure of energy $D_{kl}$. As noted in [4], a structure realizing $D_{kl}$ needs to be considered to be a possible part of the minimum free energy structure only if $f_{k}<f_{k+1}$. Otherwise, the extension of a substructure on $\left[k+1,l^{\prime }\right]$ by an unpaired base at position *k* can be chosen instead. The index position $l^{\prime }$ is determined by an evaluation of Equation (2) in the next step of the recursion.

The idea of span-restricted structures is also of interest in the context of maximum expected accuracy (MEA) methods [10,11]. The expected accuracy of a given secondary structure Ψ is the sum of its base pairing probabilities ${\hat{p}}_{ij}$, (i,j)∈Ψ, plus the sum of probabilities ${\hat{p}}_{k}:=1-\sum_{j<k}{\hat{p}}_{jk}-\sum_{j>k}{\hat{p}}_{kj}$ for the unpaired positions. Instead of treating this as a maximization problem, we mininimize $S_{ij}$, the negative of the accuracy. This highlights the similiarity of the MEA recursions with the thermodynamic folding models. MEA requires the base pairing probabolites ${\hat{p}}_{kl}$ as input. Therefore, these are computed with alternate methods, e.g., the partition function version of RNALfold [4], Rfold [5], or as the average over sequence windows that enclose the base pair of interest, as in RNAplfold [12] or LocalFold [6]. MEA models also use the weight ${\hat{p}}_{k}:=1-\sum_{j<k}{\hat{p}}_{jk}-\sum_{j>k}{\hat{p}}_{kj}$ for base *k* to be unpaired, which leads to a slight generalization of Equation (2):

(3) $$f_{k}=\min\left\{\begin{array}{c} D_{k}+f_{k+1}\\ \min_{k<l\leq k+L-1}\left[D_{kl}+f_{l+1}\right] \end{array}\right.$$

Here, an unpaired position *k* contributes $D_{k}=-{\hat{p}}_{k}$ instead of 0, and the contributions of a single-component structure that is enclosed by the pair (k,l) becomes $D_{kl}=\left(-{\hat{p}}_{kl}\right)+S_{k+1,l-1}$. The negative expected accuracy $S_{kl}$ follows the Nussinov-like [13] recursion

(4) $$S_{kl}=\min\left\{\begin{array}{c} \left(-{\hat{p}}_{k}\right)+S_{k+1,l}\\ \min_{k<j\leq l}\left[\left(-{\hat{p}}_{kj}\right)+S_{k+1,j-1}+S_{j+1,l}\right] \end{array}\right.$$

with $S_{kk}:=-{\hat{p}}_{k}$ and the convention that $S_{l+1,l}=0$ for the empty interval. The condition for local structure candidates also needs to be modified in order to account for the contribution of unpaired bases and it becomes

(5) $$f_{k}<D_{k}+f_{k+1}\;,$$

in the general case. Note that it is not necessary to store all of the values of the matrix $D_{kl}$ in the forward recursion. Instead, we backtrack at position *k* the optimal structure whenever $f_{k}$ satisfies the second alternative shown in Equation (3) and only record that structure and the values of $-{\hat{p}}_{kl}$ for the base pairs in that structure. Optimal structures contained as substructures in a larger one are omitted, as in the MFE case.

The same scheme applies to generalization of the MEA structures. In order to obtain the centroid structure [14], it suffices to consider only the base pairs with ${\hat{p}}_{kl}\geq 1/2$. The γ-centroid that was proposed in [15] is obtained by ${\hat{p}}_{k}\leftarrow 0$ and ${\hat{p}}_{kl}\leftarrow \left(\gamma +1\right){\hat{p}}_{kl}-1$. Similar expressions pertain to the estimators discussed in [16].

### 2.2. Increased Memory Efficiency by Reduced Redundancy

RNALfold reduces the memory requirements of the algorithm by only keeping a small part of the *D* (or *C*) matrices in memory, namely the range [k,k+L−1] that is necessary to evaluate Equation (2). Instead of storing the dynamic programming tables to enable backtracking, RNALfold immediately backtracks a single-component structure $\Psi_{kl_{k}}$ for *k* and then stores it on disk. Here, $l_{k}$ is the end position of the first component of an optimal structure on [k,n]. Because of the restriction on the span *L*, we know *a priori* that this structure cannot reach beyond the interval [k,k+L], i.e., $l_{k}\leq k+L$. More precisely, the end position $l_{k}$ is the value of *l*, for which the minimum in Equation (2) is attained. In the most straighforward version, the triple $\left(k,\Psi_{kl_{k}},D_{kl_{k}}\right)$ is written to disk, where triples of the form $\left(k{,}^{\prime }.^{\prime },D_{kk}\right)$ can be omitted in minimum energy directed folding, since $D_{kk}=0$, by definition, for all exterior bases.

In the simplest case, the substructure $\Psi_{kl_{k}}$ are stored in dot-parenthesis notation. Ref. [4] already noted that (together with the sequence information) these are sufficient for constructing the globally optimal secondary structure. In the case of MEA structures, $D_{kl}$ also needs to be stored explicitly. The required disk space is O(nL). It can be reduced by a considerable constant factor; however, since the energy of a given structure can be evaluated in linear time. Therefore, it suffices to store structures $\left(k,\Psi_{kl_{k}}\right)$ that are maximal in the sense that there is no $\left(k^{\prime },\Psi_{k^{\prime }l_{k^{\prime }}}\right)$, such that $\Psi_{kl_{k}}$ is proper substructure of $\Psi_{k^{\prime }l_{k^{\prime }}}$. In addition, inthe case of MEA structures, we need the proabilities ${\hat{p}}_{j}$ and ${\hat{p}}_{ij}$ for the unpaired bases and the base pairs in the candidate structure in order to be able to compute the $D_{kl}$ value for substructures that have not been explicitly stored.

In the case of MFE structures, the energy of a stored structure can be directly evaluated from the energy model. In the case of MEA structures; however, the base pairing probabilities, or more generally the derived scores, ${\hat{p}}_{kl}$ of all pairs (k,l) as well as the scores of the unpaired ${\hat{p}}_{k}$ of all unpaired positions must be available in the input. An implementation of the MEA option is forthcoming.

So far, backtracking of the global MFE or MEA structure in not available in RNALfold. Here, we close this gap. Backtracking starts from the 5’ end, i.e., from the end of the file storing the candidate fragments. For a given *k*, the task is to find $l_{k}$, such that $f_{k}=D_{k,l_{k}}+f_{l+1}$, unless $f_{k}=D_{k}+f_{k+1}$. In the latter case position *k* is unpaired and the recursion continues with k←k+1. The difficulty in the first case is that there is not necessarily an entry for *k* in the output of RNALfold. However, it suffices to consider the set of candidate structure that contain position *k*, i.e.,

(6) $$L\left(k\right):=\{\Psi_{k^{\prime },l_{k^{\prime }}}|k\in \left[k^{\prime },l_{k^{\prime }}\right]\}$$

For each Ψ in L(k), one can determine in linear time the corresponding candidate structures, as follows: (1) determine the base pair (p,q) in Ψ with the smallest value of p≥k. If the base following *q* is paired, the only candidate is the restriction Ψ[k,q]. In the case of an model with dangling ends, both Ψ[k,q] and Ψ[k,q+1] must be considered. The free energies ε(Ψ[k,q]) and ε(Ψ[k,q+1]) for these (explicitly given) sub-structures can be evaluated in linear time. Then one has to check, for Ψ∈L(k) and $q^{\prime }=q$ and, in the case of dangling ends, also $q^{\prime }=q+1$, where the structure satisfies

(7) $$f_{k}=\varepsilon \left(\Psi \left[k,q^{\prime }\right]\right)+f_{q^{\prime }+1}\;.$$

The evaluation continues at position $k\leftarrow q^{\prime }+1$, where $q^{\prime }$ is the first alternative for which equality is found in Equation (7). The backtracking method is also applicable without change to computations with constrained structures [17], since it only relies on the fact that a structure fragment is available in the output of the forward recursion that satisfies Equation (7).

### 2.3. Performance Analysis and Implementation

A naïve estimate of the CPU requirement that is required for backtracking yields an upper bound of $O(nL^{2})$, since |L(k)| contains, at most, O(L) entries of size at most O(|L|), each of which certainly can be evaluated in linear time. However, it is not necessary to construct the list L(k) “from scratch” in each step. Instead, for each position *k*, at most one additional entry $\Psi_{k,l_{k}}$ is added to the list, and every other entry can be “edited” after it has been processed for position k−1 by removing from the 5${}^{\prime }$ end a leading unpaired position or the base pair $\left(k-1,l_{k-1}\right)$, respectively, as well as any structures trailing $\left(k,l_{k+1}\right)$. Assuming that the dot-parenthesis structure has been converted into an ordered list of base pairs, the effort to adjust a list entry requires only constant time to obtain $\Psi [k,l_{k}]$ from $\Psi [k-1,l_{k-1}]$. Thus, the evaluation of the energies or MEA scores requires only constant effort for each position that is removed. Because the total size of the stored structures is bounded by O(nL), the total effort for backtracking is also bounded by O(nL).

**Figure 1:**
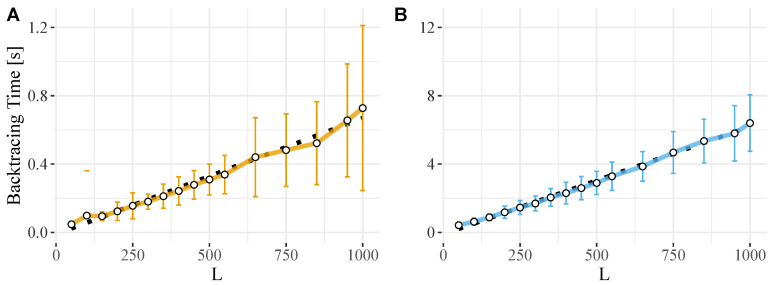
CPU requirements for backtracking the MFE structure in RNALfold. The performance of the implementation ViennaRNA conforms to a linear dependence of backtracking time per nucleotide with base pair span *L*, i.e., $t_{\mathrm{CPU}}∼\sum nL$. Shown is the computational overhead for different window sizes *L* that were obtained from averaging over 100 random sequences of length (**A**) 10,000 nt and (**B**) 100,000 nt. Error bars denote the standard deviation within the sets of 100 runs, and a linear fit is depicted by a dashed black line.

The backtracking algorithm is integrated into the ViennaRNA package and it si available for RNALfold with the command line option -b/--backtrack-global. Empirical tests, see Figure 1, show that the implementation conforms to the theoretical O(nL) performance bound.

### 2.4. New Options in RNALfold

In order to better support genome-wide screens for structured RNA elements, we added options to filter structural components, which is, maximal substructures that are enclosed by a base pair. This is motivated, in particular, by the observation that the secondary structures of many structured small ncRNAs are more stable than expected from their sequence composition [18,19,20]. This effect is particularly pronounced for miRNAs [21]. This relative stabilization for a candidate sequence *x* is conveniently quantified as a *z*-score, $z\left(x\right):=\left(f\left(x\right)-\bar{f}\right)/\sigma_{f}$, where f(x) is the folding energy of *x*, and $\bar{f}$ and $\sigma_{f}$ denote the mean and standard deviation of the folding energies of an ensemble of sequences with the same sequence composition. Computing z(x) can be viewed as a regression problem in terms of parameters that specify the composition of *x* [22]. Here, we use the SVM model of RNAz 2.0 [23], which explicitly depends on dinucleotide frequencies.

The regression approach, in contrast to shuffling, allows for a very fast (and deterministic) computation of the *z*-score $z_{kl}^{C}$ of the sub-structure that is enclosed by the base pair (k,l). This can be used in two ways to restrict the predicted structure to components with $z_{kl}^{C}$ below a user-defined threshold $z^{*}$: (1) one can already restrict the forward recursion Equation (2) to base pairs enclosing components with a sufficiently negative *z*-score (pre-filter), and (2) one can suppress components with an insufficient *z*-score in the backtracking step (post-filter). The two methods are *not* equivalent. For example, in case (2), it is possible that larger component structure with better MFE but below-threshold *z*-score is computed at the expense of a smaller structure with better *z*-score.

Both of the methods have been implemented in RNALfold. The restriction of the forward recursion is accessed with the new option RNALfold --zscore-pre-filter. Backtracking is then unaffected by the restriction to components that surpass the *z*-score threshold. As an alternative, filtered backtracking of the unmodified RNALfold output (post-filter) is performed in default mode, i.e., whenever this option is omitted. For combinations of *z*-score filtering and backtracking of global MFE structures, as described in Section 2.1; however, RNALfold automatically activates the newly implemented restriction of the forward recursion. This is due to the fact that all of the structural alternatives that constitute the global solution are required for successful backtracking. Furthermore, RNALfold defaults to omit locally optimal structures if they are constituents of another, larger structure with less free energy. This might be undesirable for predictions with z-score filtering, as the substructure may exhibit a lower z-score than the larger, enclosing structure. The novel option RNALfold --zscore-report-subsumed can be used in order to alleviate this effect.

## 3. Application: Scanning Genomes for “Hyper-Stable” RNA Structures

Some of the early surveys for ncRNAs used GC content and folding energy as indicators of structured RNAs. This approach was successful in particular in A/T-rich genomes of hyperthermophiles, such as *Methanococcus jannaschii* or *Pyrococcus furiosus* [24]. The extended version of RNALfold now makes it particularly easy to scan genomes for unexpectedly stable local structure.

**Figure 2:**
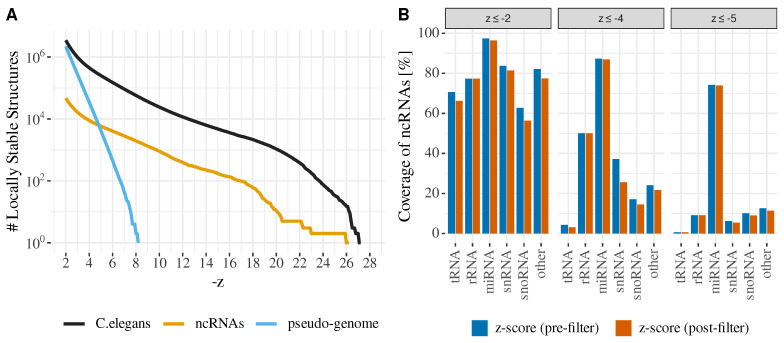
Predictions on the *C. elegans* genome. (**A**) Cumulative *z*-score distribution of predicted blocks with pre-filtering and default *z*-score threshold of z≤−2.0 and window length L=150. Shown are all predicted blocks for the entire *C.elegans* genome (black line) and those that sufficiently overlap with annotated *ncRNAs* (yellow line). The *pseudo-genome* (lightblue line) denotes blocks that were predicted on a di-nucleotide shuffled *C. elegans* genome. (**B**) Comparison of prediction coverage (L=150) of the two *z*-score filter methods. Shown is the percentage of annotated ncRNAs that are sufficiently overlapped by the predicted locally stable structures at different *z*-score filter thresholds.

As a show-case application, we screened the genomes of nematode *Caenorhabditis elegans* (Assembly WBcel235, Genome Assembly ID GCA_000002985.3) for highly stable component structures. For a given cut-off value $-z^{*}$, we recorded all of the components with a *z*-score $z\leq -z^{*}$ and compared the results to the ncRNA annotation available at Ensembl Release-101. In terms of the annotated elements, we found that there are only marginal differences between the two alternative strategies for z≤−2, see Figure 2. As expected, recall decreases in a class-specific manner as the *z*-scores become more negative. In particular, microRNAs persist longer than other classes of ncRNAs.

A comparison of the real data with a pseudo-genome that are generated by dinucleotide-shuffling [25] shows that the number of local structures that are more stable than a given *z*-score threshold $z^{*}$ decreases exponentially with $-z^{*}$, as in Figure 2A. The real data only follow the same distribution for small $-z^{*}$, but they show a tail with a smaller slope for large values of −z. This indicates that the genome is enriched in “hyperstable” RNA structures. A comparison of the distribution with annotated ncRNAs (including long non-coding RNAs, which are not expected to be particularly heavily structured over their whole length) suggests that this tail, indeed, corresponds to structured RNAs. The data also indicate that the vast majority of the approximately 57,000 “hyper-stable” elements with *z*-scores below −8 have remained unannotated. Approximately 41,000 (>70%) of those elements are predicted within low-complexity and repeat regions, as detected by RepeatMasker, while only 222 partially overlap with annotated coding sequences. For the former, we find that a large number of the predicted hyper-stable elements overlap with repeat classes/families that are annotated as DNA transposon (11,561), Simple Repeats (7977), and Satellite (7832). Long terminal repeats (LTR) are overlapped by 417 hyper-stable elements, while general low complexity regions, LINEs, and SINEs are overlapped by 153, 55, and 14, respectively. Low complexity repeats have previously been described to form highly stable structures and they have been studied, in particular, in the context of triplet repeat expansion diseases [26].

We further compared our predictions for the A/T-rich genomes of hyperthermophiles against the 32 candidate ncRNAs of length 49–238 nt listed in Klein et al. [24]. Here, we find that using default settings (L=150, $-z^{*}=-2.0$), both approaches, pre- and post-filter, predict locally stable elements that overlap with at least 10% for virtually all of the candidate ncRNAs. The only exception is Mj8, which is not detected by the post-filter method. When requiring 50% overlap, the pre- and post-filter approach detects 21 and 22 out of a total of 22 candidate ncRNAs for *Pyrococcus furiosus*, respectively. For *Methanococcus jannaschii*, pre- and post-filter yields nine and eight of a total of 10 candidate ncRNA loci. With 12 and 10 out of 22 candidate ncRNA loci in *P. furiosus* for pre- and post-filter, respectively, approximately one half is fully overlapped (100%) by our predictions. This is similar to the predictions for *M. jannaschii*, where we find seven and four out of 10 candidate ncRNA loci, respectively. When we increase the window length to L=250 to accomodate the lengths of the queries, all 10 candidate ncRNAs in *M. jannaschii*, and the majority (20 for pre-filter, 18 for post-filter) of the 22 candidate ncRNAs in *P. furiosus* are detected with an overlap of 100%. For the elements that fully overlap with the candidates, the majority of *z*-scores is larger than −3.0, where the lowest *z*-score found is about −4.8. Still, among our pre-filtered predictions are a further 927 (*P. furiosus*) and 3007 (*M. jannaschii*) locally stable structures with z<−4.8, which account for 2.5% and 4.5% of the genomic DNA, respectively. A closer investigation reveals that approximately 29% (*P. furiosus*) and 22% (*M. jannaschii*) of these predicted elements overlap, at least in part, with other annotated ncRNAs, including rRNAs amd tRNAs. On the other hand, about 35% and 27% partially overlap with protein coding regions in the two genomes, respectively. This leaves 338 elements at 136 distinct non-coding loci in *P. furiosus* and 1609 elements at 287 loci in *M. jannaschii* as novel ncRNA candidates.

## 4. Conclusions

In this contribution, we have described an algorithm to reconstitute the MFE RNA secondary structure with limited base pair span from locally optimal structures. The method is applicable to effectively arbitrarily long RNA sequences and it closes the gap in the current toolkit that is provided by the ViennaRNA package. Arguably, the exact computation of such span-restricted MFE is of limited interest, since most RNA molecules of practical interest do not exceed the length range tractable without span restrictions. Furthermore, RNALfold and RNAplfold are primarily intended to scan entire genomic regions and provide local information, i.e., tasks for which local predictions that were provided by RNALfold could be used. However, the overlapping nature of these predictions is inconvenient, in particular, in the context of annotation, where one would like a partition of the input sequence into disjoint local structures. In order to become interpretable, the output of RNALfold therefore requires some form of postprocessing to reconcile overlapping local structures. The span-restricted MFE structure by construction consists of a partioning into pairwise disjoint components, i.e., base pair enclosed domains. Because the backtracking procedure that is described here has a running time of O(nL), it is asymptotically optimal in the sense that postprocessing tools cannot be much faster, since the total amount of output of the forward recursion is also of size O(nL).

The new backtracking functionality makes it easy to scan genome-scale data set for unusually stable structure. First and foremost, this provides a potentially useful pre-filter for other, more computationally demanding methods that search for specific types of non-coding RNAs, in particular microRNAs. However, Figure 2 also indicated that there is a large number of “hyper-stable” secondary structure elements that deserve more attention in their own right. The bulk of the hyperstable structures in *C. elegans* falls into repetitive elements. Earlier studies of structured ncRNAs explicitly excluded repetitive DNA. Our results suggest that these deserve more detailed attention in future research.

## Data Availability

The software described in this contribution is available as part of the ViennaRNA package https://www.tbi.univie.ac.at/RNA/.

## Abbreviations

The following abbreviations are used in this manuscript:

MFE	Minimum free energy (secondary structure)
miRNA	microRNA
ncRNA	Non-coding RNA
MEA	Maximum expected accuracy (secondary structure)
TLA	Three letter acronym
